# Chest wall tumor and chest wall reconstruction surgery using Tahalele's method

**DOI:** 10.1186/1749-8090-8-S1-O232

**Published:** 2013-09-11

**Authors:** PL Tahalele

**Affiliations:** 1 Department of Surgery Division of Cardiothoracic and Vascular Surgery School of Medicine Airlangga University - Dr. Soetomo General Hospital Surabaya, Indonesia

## Background

Chest wall tumors are a heterogenous group of lesions that provide an interactive therapeutic and reconstructive challenge for surgeons.

## Purpose

To show our experience in chest wall surgery today which calls for complete resection of chest wall tumor, reconstruction of the thoracic defect with semirigid wire and covered by soft tissue.

## Methods

Retrospective study from 1986 to 2012 (26 years) at The Division of Thoracic and Cardiovascular Surgery Dr. Soetomo Hospital Hospital Surabaya, reporting 101 cases of chest wall tumor excision and chest wall reconstruction using Tahalele’s method.

## Results

64 patients with primary neoplasm (63.37 %) and 37 patients with secondary tumor (36.63%). There were 30 patients (29.70%) with benign tumor and 71 patients (70.29%) with malignant tumor. Primary tumor consist of chondroma (13 patients,12.87 %), osteoma (14 patients, 13.86 %), benign toratoma (one patient,0.99 %), chondro sarcoma (4 patients ,3.96%), osteo sarcoma (22 patients, 21.78%), soft tissue sarcoma (6 patients, 5.94%), Ewing sarcoma (one patient,0.99%), rhabdomyosarcoma (3 patients, 2.97 %). Metastatic tumor consist of metastatic breast cancer in 22 patients (21.78%), metastatic malignant teratoma in 2 patients (1.98%), matastatic thyroid cancer in 13 patients (12.87%). In the follow-up four years after the operation, no operative mortality. Hospital mortality in 2 patient (1.98%). Local recurrence in 4 patient (3.96%). Five patients with partial flap necrosis (4.95%). No flail chest complication (0 %). Infection rate after operation 3 patient (2.97%). Average LOS after the operation 7-21 days. Loss of follow-up were 58 patients (57.43%), and 52 patients survive (51.48%).

## Conclusion

All the patients were satisfied with this method, complete resection of chest wall tumor, reconstruction of chest wall defect with semirigid wire (Tahalele’s method) and covered by soft tissue Chest physiotherapy before operation play a significant rule.

